# The Bruit De Diable, or Anæmic Murmurs

**Published:** 1872-06-01

**Authors:** 


### THE BRUIT DE DIABLE, OR ANÆMIC MURMURS.

### A LECTURE BY DR. A. DUCHEK, PROF. OF GENERAL PATHOLOGY IN THE UNIVERSITY OF VIENNA.

(*From Notes by Karl Wavlik.*)

There are to be found murmurs in the heart and the veins, which do not arise from any substantial organic changes in the heart or vessels, as for instance in men and animals exhausted by loss of blood and attendant anæmia. In the same degree as the loss of blood and the paleness of the skin proceed, these murmurs appear in the heart and the veins of the throat. In severe cases if we touch the jugular vein with the finger we perceive at once a continued vibration like that of a string. On pressure on the upper part of the jugular vein, both the perception of the vibration and the acoustic evidence of a murmur disappear, proving it to be merely venous.

The formation of the heart sounds is modified by two circumstances : the condition of the valves of the heart and the vessels on the one hand, and the pressure produced by the blood stream upon them on the other. As we have in such cases of anæmia no symptoms permitting us to diagnose changes in the vibrating mediums, we are induced to seek for the origin of these sounds in the causes themselves which produce vibration, viz., the pressure of the blood. In the disorders attended by these murmurs (as anæmia, chlorosis and loss of blood), the propulsive power of the heart is unchanged, but its contractions are more frequent, while the blood is either diminished in quantity or weakened in quality. The pressure exercised by it in the circulation is also diminished, so that the impulse given by the blood column is not sufficient to develop in the valves, or in the arterial walls, the amount of tension necessary for the formation of the sound, which under normal circumstances makes its appearance in these vibrating mediums, but in these cases is lowered into the acoustic impression of a murmur.

In this way we explain the origin of the murmurs in the heart. It is more difficult, however, to trace its cause in the jugular vein. Some have attributed it to the arteries, but this is contradicted by its disappearance on pressure on the jugular vein, as well as by its continuity. These sounds are modified in two ways. They grow stronger at regular intervals, being reinforced by the sounds of the carotids ; we call these *moments of reinforcement*. Secondly, the murmurs are increased or diminished according to the force and rapidity of the respiration.

In the heart we attribute all sounds and murmurs to the action of *vibrating membranes*. But for these murmurs in the veins we cannot suppose that they are caused by a *whirl* of *blood*, nor by the pushing of the blood stream against a narrow entrance. Behind the insertion of the sterno-cleido-mastoideus the jugular vein is wider than elsewhere, forming the bulbus, and beneath this bulbus, in the narrowest part of the vein, are valves. These valves have a most important influence upon the entire circulation. If the pressure in the thorax is too great they are approximated and thus oppose the further entrance of the blood. The part of the veins above them becomes, consequently, dilated just as in cases of stagnation in the heart, the jugular vein is distended and the jugulum grows gradually more shallow.

The venous murmurs then arise from the vibrations of these valves caused by the impulse of the blood. When the blood flows slowly the impulse is too weak and no murmur is to be heard. The murmur arises when the valves are half opened and put into vibration by a sufficient rush of the blood.

Another peculiarity is to be noted. The bulbus is attached to the clavicle, behind the sterno-clavicular articulation, and thus being stretched out, the flow of blood is facilitated. If the pressure in the thorax is increased by valvular failures, emphysema of the lungs, etc., the stream flows slowly, and therefore we do not find these murmurs attending disorders of the intra-thoracic organs. Another consequence of this fact is, that these murmurs disappear in anæmic persons when they become affected by pneumonia or exudative pleurisy, reappearing again however on recovery. The two necessary requisites then, for the production of the murmur are a speedy circulation, and a normal pressure of blood in the thorax.

This view also affords us an explanation of the fact of the murmurs growing stronger when the respiration is more hurried. The thorax being strongly dilated, the blood rushes into it in more forcible currents, thus strengthening the acoustic impression. When the respiration is impeded, or is forced, the circulation loses the necessary rapidity, and the vibrations become inaudible.

These murmurs are very rarely to be found in connection with mechanical changes in the heart. They accompany similar murmurs at the aorta, which are always systolic, never diastolic. They may perhaps be diastolic in the most severe disorders of this kind, where it is impossible to distinguish whether there is only one continued murmur, or whether the systolic and diastolic murmurs meet and run into one another.

---

GRANULATIONS OF THE CONJUNCTIVA TREATED BY ELECTRICITY.—Two methods of treating granulations of the lids by electricity, are noticed. Dr. Kohn, of Berlin, employed a battery of one element. Having protected the eye with Jaeger's plates, he placed the heated platinum wire on the eyelid. Dr. Schivardi employed a Bunsen battery of two elements. The negative pole is applied to the inverted surface of the upper lid by means of a buttoned sound. The positive pole is applied to the nucha by means of a sponge saturated in salt water. The session is of about ten minutes duration. Remarkable success is claimed.

